# DEPRESSIVE SYMPTOM TRAJECTORIES AND COGNITIVE FUNCTION AMONG OLDER COUPLES: A DYADIC PERSPECTIVE

**DOI:** 10.1093/geroni/igac059.908

**Published:** 2022-12-20

**Authors:** Dexia Kong, Peiyi Lu, Jean Woo, Mack Shelley

**Affiliations:** The Chinese University of Hong Kong, Hong Kong, Hong Kong; Columbia University, New York, New York, United States; Faculty of Medicine, Hong Kong, Hong Kong; Iowa State University, Ames, Iowa, United States

## Abstract

Despite the well-documented health interdependence in the spousal context, empirical evidence on how psychological wellbeing of one’s partner might affect one’s cognitive function remains limited. Using dyadic data, the objective of this study is to examine trajectories of depressive symptoms and associated cognitive function outcomes among U.S. older married couples. Longitudinal Health and Retirement Study data (2004-2016) were used (N=6,289 heterosexual couples). Latent class growth analysis characterized depressive symptom trajectories for wives and husbands, separately. Structural equation models examined the actor and partner effects of depressive symptom trajectories on cognitive function in 2016 after adjusting for covariates. Four distinct depressive symptom trajectories were identified, including persistently low (wives: 73.61%; husbands: 79.59%), increasing (wives: 8.60%; husbands: 8.27%), decreasing (wives: 12.80%; husbands: 8.32%), and persistently high (wives: 4.99%; husbands: 3.81%). Compared to the low trajectory, increasing and high depressive symptom trajectories were associated with poorer cognitive function for wives and husbands (β_(wife,increasing,actor)=-0.92,95%CI=-1.30,-0.54; β_(wife,high,actor)=-0.71,95%CI=-1.19,-0.23; β_(husband,increasing,actor)=-0.81,95%CI=-1.16,-0.45; β_(husband,high,actor)=-1.20,95%CI=-1.78,-0.63). Notable gender discrepancies in partner effects were observed. Specifically, wife’s depressive symptom trajectories were not associated with husband’s cognitive function (P>0.05). However, husband’s decreasing depressive symptom trajectory was linked to wife’s better cognitive function. One’s own depressive symptom trajectories predicts his/her own cognitive function. Specifically, older adults with increasing and persistently high depressive symptoms over time may experience poorer cognitive function, and thereby warrant additional policy and clinical attention. Psychosocial interventions targeting depressive symptoms among older men may be beneficial to their spouses’ cognitive function. Future studies need to validate such gender differences.

